# Continuous renal replacement therapy and transplant-free survival in acute liver failure: protocol for a systematic review and meta-analysis

**DOI:** 10.1186/s13643-020-01405-7

**Published:** 2020-06-16

**Authors:** Andrea M. Robinson, C. J. Karvellas, Joanna C. Dionne, Robin Featherstone, Meghan Sebastianski, Ben Vandermeer, Oleksa G. Rewa

**Affiliations:** 1 Department of Critical Care Medicine, University of Alberta, 2-124 Clinical Sciences Building, 8440 112th St NW, Edmonton, AB T6G 2B7 Canada; 2 Division of Gastroenterology, Department of Critical Care Medicine, University of Alberta, 1-40 Zeidler Ledcor Building, 8540-112 St, Edmonton, AB T6G 2X8 Canada; 3 Department of Medicine, Division of Critical Care, McMaster University, Hamilton, Canada; 4 Alberta SPOR Knowledge Translation Platform, University of Alberta, Edmonton, 4-486D Canada; 5 Department of Health Research Methods, Evidence, and Impact, McMaster University, Hamilton, Canada; 6 Alberta Research Centre for Health Evidence (ARCHE), Department of Pediatrics, University of Alberta, Edmonton, Canada; 7 Edmonton Clinic Health Academy (ECHA), University of Alberta, 11405-87 Ave, Edmonton, AB T6G 1C9 Canada

**Keywords:** Acute liver failure, CCRT, Hyperammonemia, Transplant-free survival

## Abstract

**Background:**

Acute liver failure is a rare syndrome with significant morbidity and mortality, particularly in absence of transplantation as a rescue therapy. An important mechanism contributing to mortality is hyperammonemia which drives cerebral edema and raised intracranial pressure. Multiple therapies for managing hyperammonemia have been trialed. Continuous renal replacement therapy is effective in treating hyperammonemia in other disease states (notably inborn errors of metabolism). Its efficacy in acute liver failure has been suggested but further investigation is required to prove this. The objective of this systematic review will be to determine the efficacy of continuous renal replacement therapy in patients with acute liver failure and its effect on mortality and transplant-free survival.

**Methods:**

MEDLINE, EMBASE, Web of Science, and Cochrane Database will be searched. Identified studies will include all patients with acute liver failure in a critical care unit treated with continuous renal replacement therapy. Primary outcome will be effectiveness of ammonia clearance and mortality. Patients treated with any other modality of ammonia lowering therapy (such as plasma exchange or Molecular Adsorbent Recirculating System) will be excluded. Narrative synthesis of the identified studies will occur and if clinical homogeneity is identified, data will be pooled for meta-analysis using a DerSimonian-Laird random effects model.

**Discussion:**

We present a protocol for a systematic review seeking to establish a link between transplant-free survival in acute liver failure and the use of continuous renal replacement therapy. Given the anticipated paucity of literature on this subject, both narrative and quantitative syntheses are planned.

**Systematic review registration:**

(PROSPERO) CRD42019122520, registered April 16, 2019.

## Background

Acute liver failure (ALF) is defined by the American Association for the Study of Liver Disease (AASLD) as a new coagulopathy (with International Normalized Ratio [INR] ≥ 1.5) and any degree of encephalopathy in a patient with new onset liver failure (< 26 weeks) [1]. It is a rare entity with an estimated incidence of between 1 and 6 cases per million people per year [2]. It is distinct from both chronic liver failure and acute on chronic liver failure [3]. Its defining characteristic is the absence of premorbid liver disease [4] and thus the complications of long-term portal hypertension. There are numerous etiologies, including viral and autoimmune causes, but the most common is drug-induced liver injury of which acetaminophen is the most common, accounting for 46% of all acute liver failure in some studies [5]. Although rarer than other forms of chronic liver failure, it nonetheless caries a high burden of morbidity and mortality [4, 6, 7]. Multiple therapies have been investigated, including Molecular Adsorbent Recirculating System (MARS) and plasma exchange, which either have not led to improved survival or require specialized centres and intensive resources to administer [8, 9]. Importantly, one current rescue therapy exists in the form of liver transplantation. However, liver transplantation requires lifelong immunosuppressive therapy and leads to associated transplant related morbidities, including infection risk, secondary diabetes mellitus, and osteoporosis due to the immunosuppression [10]. The availability of donor livers for transplantation continues to be limited, particularly in countries of opt-in donation such as Canada and the USA [11]. Further, determining candidacy for liver transplantation is a complex process and not all patients are deemed eligible, on the basis of co-morbidities, social circumstances, or other factors. Overall survival in acute liver failure is as high as 75%, and transplant-free survival remains significantly lower at 56% [4, 6]. Given this survival discrepancy of nearly 25%, there is a need for a deeper understanding of the pathophysiology of acute liver failure and therapies that will help bridge to transplantation or obviate the need for transplantation.

Mortality and morbidity in acute liver failure are driven by multiple mechanisms, including vasodilator shock and multi-organ failure. An important complication of ALF is hepatic encephalopathy which is with associated intracranial hypertension [12]. In contrast to chronic liver failure where manifestations of metabolic encephalopathy are often limited to a decreased level of consciousness [13, 14], acute liver failure also leads to increased cerebral edema, raised intracranial pressure, and herniation risk [15, 16]. Elevated serum arterial ammonia level correlates with increased risk of cerebral edema in acute liver failure [17] although a specific cutoff value has not clearly been identified [18]. Intracranial hypertension has been associated with increased mortality and decreased transplant-free survival [7, 19].

Multiple strategies have been trialed to decrease serum ammonia and improve cerebral edema including the use of hypertonic saline [1, 20], targeted temperature management [21], and the use of plasma exchange [8, 22]. One such strategy is the use of continuous renal replacement therapy (CRRT).

The use of CRRT for clearance of hyperammonemia and improvement in cerebral edema is best described in the pediatric literature where it is used for management of inborn errors of metabolism [23, 24]. However, the mechanism by which ammonia accumulation leads to cerebral edema is comparable between the two populations [16].

CRRT also plays an important role in management of acute kidney injury (AKI). According to one study, this can occur in up to 70% of patients with ALF, and 30% requires renal replacement therapy, increasing both morbidity and mortality [25]. Thus, its use in patients with acute liver failure and co-existing AKI is not uncommon. However, its role in ammonia clearance as a mechanism for decreasing or preventing cerebral edema is not established. The use of CRRT in acute liver failure has been described in some works and shown improved outcomes in patients with renal injury/failure undergoing liver transplantation [26]. Some evidence points to its successful use as a bridge to transplantation in the pediatric population [27].

The primary objective of this systematic review will be to determine the efficacy of CRRT in patients with acute liver failure and its effect on mortality and transplant-free survival. Our hypothesis is that the use of CRRT in patients with acute liver failure may lead to improved cerebral edema and improved transplant-free survival. The main research question being address is “In patients with acute liver failure, does the use of continuous renal replacement therapy improve transplant-free survival compared to usual care?” This will help guide future research inquiries including observational and experimental trials.

## Methods

### Study design

The present protocol has been registered within the PROSPERO database (registration ID: CRD42019122520). The present study protocol is being reported in accordance with the reporting guidance provided in the Preferred Reporting Items for Systematic Reviews and Meta-Analyses Protocols (PRISMA-P) statement [28] (see PRISMA-P checklist in Additional file 1). The proposed systematic review will be reported in accordance with the reporting guidance provided in the Preferred Reporting Items for Systematic Reviews and Meta-analyses (PRISMA) statement [28] and the Meta-analysis Of Observational Studies in Epidemiology (MOOSE) reporting guideline [29].

### Search strategy

The search strategy has been developed in consultation with a research librarian from the Alberta SPOR Support Unit and independently reviewed by a second librarian (see Additional file 2). The following databases will be used: MEDLINE, EMBASE, Cochrane Library, and Web of Science. In addition, grey literature citations will be searched using ClinicalTrials.gov and the Conference Proceedings Citation Index. Where data is not available in the published literature, authors will be contacted to request additional information. Databases will be searched from 1990 to 2020, as this is shortly after the first published description of veno-venous continuous renal replacement therapy in 1982 [30].

### Inclusion criteria

Studies will be selected according to the following criteria: participants, interventions and comparators, outcome(s) of interest, and study designs. Given the anticipated paucity of high quality randomized controlled trials, all study designs, including randomized trials, cohort studies, and case control studies, will be included. Case reports and series will not be including given the general lack of a comparator arm in such studies. Studies from all languages will be considered, so long as English translation is available. Data presented from conference abstracts will also be included as well as that published in journals. The inclusion criteria for this study are adult patients (over 18 years of age) admitted to an intensive care unit with a diagnosis of acute liver failure. Any etiology of acute liver failure will be considered. The intervention sought is the use of continuous renal replacement therapy while the comparator is usual care without renal replacement therapy. Acute liver failure will be defined in accordance with the AASLD guidelines as liver disease of less than 26 weeks’ duration and the presence of coagulopathy (international normalized ratio [INR] ≥ 1.5) as well as any degree of encephalopathy [1]. CRRT may be used for one or more indications (i.e., AKI and fluid management), including management of hyperammonemia.

### Exclusion criteria

Case studies and case series will be excluded, as will any existing reviews, meta-analyses, commentaries, or opinion pieces. Exclusion criterion for this review is the presence of chronic liver failure more than 26 weeks’ duration prior to ICU admission. Studies will also be excluded if another form of ammonia lowering therapy (e.g., Molecular Adsorbent Recirculating System [MARS] or plasma exchange) was used in addition to CRRT.

### Intervention and comparator

The studied intervention is the use of continuous renal replacement therapy, employed at any point during critical illness. All forms of veno-venous renal replacement therapy (including hemofiltration and hemodiafiltration) will be considered. The comparator is usual care which includes all drugs and supportive care used in the management of acute liver failure. In keeping with exclusion criteria above, other forms of ammonia lowering therapy (such as MARS and plasma exchange) are not considered to be usual care.

### Outcome measures

For the acute liver failure group, the primary outcome of interest is transplant-free survival and mortality at 21 days. Secondary outcomes are reduction in serum ammonia levels over 72 h following admission or initiation of CCRT, persistence of multi-organ failure, degree of metabolic encephalopathy (using West Haven grade over 72 h), hospital or ICU length of stay, and use of life sustaining therapies.

### Risk of bias assessment

Selected studies will be assessed by two independent authors using the Newcastle Ottawa Scale for observational studies (likely the majority of identified studies) [31]. Any included randomized controlled trials will be assessed for methodological quality using the Cochrane Collaboration’s Risk of Bias (RoB) 2.0 tool for randomized controlled trials [32].

### Study selection and data extraction

Identified studies will be reviewed by two independent authors. Where differences exist, consensus will be sought through discussion. First, titles and abstracts will be screened for eligibility criteria. Once primary studies are screened, included studies will proceed to full-text review and will be assessed based on criteria outlined above. Authors of selected studies will be contacted if necessary to provided missing and relevant data. A flow chart showing studies included or excluded at each stage of the process will be provided.

Data will be extracted using a standardized form generated a priori by the authors. Extracted data will include wherever available a number of variables. With respect to patient characteristics, age, etiology of liver failure, stage of encephalopathy, number of patients study inclusion and exclusion criteria, and geographic location will be recorded. Frequency, dose, and duration of continuous renal replacement therapy will be extracted from the intervention. Any co-interventions used (such as hypertonic saline) in either the intervention or comparator group will be recorded. With respect to outcomes, the method and timing of assessment and any adverse reactions will be included. Finally, outcomes related to mortality, transplant-free survival, and ammonia levels pre and post treatment will be extracted. Where reported, number and modality of required life support interventions and ICU and hospital length of stay will be extracted. At a study level, any reported funding sources, the process through which consent was obtained and presence of ethics board approval, will be recorded.

### Data synthesis

Data will first be analyzed qualitatively in the form of summary table and narrative synthesis. This will be used to assess heterogeneity of interventions and outcomes in order to gauge the feasibility of meta-analysis. Heterogeneity will be evaluated using the I-squared statistic. If sufficient clinical homogeneity is identified among the studies, data will be pooled for meta-analysis using a DerSimonian-Laird random effects model. Binary outcomes (i.e., survival) will be pooled using risk ratios while continuous outcomes (i.e., length of stay) will be pooled using mean differences. All estimates will be reported with 95% confidence intervals.

### Meta-biases

The primary anticipated source of meta-bias will be a publication bias given the small number of observational trials on this subject. Clinicaltrials.gov will be searched for evidence of unpublished or abandoned trials, which will be assumed to be negative. If any individual meta-analysis contains more than 8 studies, small study bias (publication) bias will be evaluated using a funnel plot. Finally, outcome measures extracted from these studies will be gauged using the Grading of Recommendations, Assessment, Development, and Evaluation (GRADE) classification.

## Discussion

Acute liver failure is a rare form of liver failure with nonetheless important impacts on morbidity and mortality. Although survival can be as high as 75%, this is heavily influenced by the use of liver transplant as a rescue therapy, a procedure associated with both immediate and chronic complications. Transplant-free survival is significant lower, at around 55% [4, 5]. Transplantation remains the definitive cure but is associated with the risk of many complications. These include the acute peri-operative morbidity and mortality as well as the long-term risk of graft rejection and chronic immunosuppression. Identification of an effective alternative is therefore of benefit to this patient population.

Previously studied investigational therapies, including MARS and hypertonic saline, have not clearly shown a mortality benefit [9, 20]. Therapeutic plasma exchange has shown some promise in at least one randomized controlled trial [8]. The proposed beneficial mechanism rests on the removal of inflammatory cytokines and the replacement of plasma factors to attenuate the inflammatory response as a means to reduce cerebral edema. It is feasible, however, that CRRT can also serve to remove harmful toxins, particularly ammonia. If successful, this is advantageous over TPE as it is a more cost-effective therapy, does not require specialized centres to deliver, and is does not carry the same risks of blood product exchange that TPE does. There is need for investigation of therapeutic modalities to improve transplant-free survival in acute liver failure. A major anticipated limitation of the present review is the paucity of methodologically robust clinical trials investigating the hypothesis of interest. It is the expectation of the study authors that identified trials will primarily be observational in nature, thus limiting the applicability of the conclusions drawn. As the primary purpose of this review is to guide future investigational research, this data will hopefully lend credence to the hypothesized mechanism and support further experimental research in the acute liver failure population. A second limitation is the inclusion of all etiologies of liver failure in the review. It is feasible that the natural history of different etiologies (acetaminophen overdose vs. viral disease) has different natural histories and that this would influence transplant-free survival. Given that this is a disease of overall low incidence, this is inevitable at the present time, but etiology of liver failure will be documented and considered as a variable influencing outcomes. This systematic review will synthesize available literature on the use of CRRT for clearance of hyperammonemia in ALF and aim to establish a link between this and transplant-free survival. Results will be presented at conference proceedings in poster or oral presentations. The final manuscript will be submitted to a peer-reviewed journal for publication. Any major changes to the protocol following publication of this protocol will be decided by consensus among the authors and included in the final manuscript. This systematic review and meta-analysis will help inform future trial development regarding the effectiveness of CRRT for ALF.

## Supplementary information

**Additional file 1:.** PRISMA-P 2015 Checklist

**Additional file 2:.** Sample MEDLINE search strategy

## Data Availability

Not applicable

## Abbreviations

AASLD: American Association for the Study of Liver Disease
AKI: Acute kidney injury
ALF: Acute liver failure
CRRT: Continuous renal replacement therapy
ICU: Intensive care unit
INR: International normalized ratio
MARS: Molecular adsorbent recirculating system
PRISMA-P: Preferred Reporting Items for Systematic Reviews and Meta-Analyses Protocol
PROSPERO: International Prospective Register of Systematic Reviews
TPE: Therapeutic plasma exchange
