# Supplementary material for: Continuous renal replacement therapy and transplant-free survival in acute liver failure: protocol for a systematic review and meta-analysis
Source: Syst Rev. 2020 Jun 16;9:143. doi: 10.1186/s13643-020-01405-7 (PMC7296967; doi:10.1186/s13643-020-01405-7)
Supplement: Supplementary file 2 — Additional file 2:. Sample MEDLINE search strategy [file 13643_2020_1405_MOESM2_ESM.docx]

**Table 1. Sample MEDLINE search strategy**

| 1 exp Hemofiltration/ (6478)  2 Renal Dialysis/ (86103)  3 Renal Replacement Therapy/ (4930)  4 (dialys* or haemo-dialys* or haemodialys* or hemo-dialys* or hemodialys*).tw,kf. (149329)  5 (haemo-diafiltrat* or haemo-filtrat* or haemodiafiltrat* or haemofiltrat* or hemo-diafiltrat* or hemo-filtrat* or hemodiafiltrat* or hemofiltrat*).tw,kf. (6573)  6 ((kidney* or renal) adj1 replacement adj2 (therap* or treat* or support*)).tw,kf. (11761)  7 RRT.tw,kf. (3805)  8 or/1-7 [Combined MeSH & text words for RRT] (178400)  9 (24h or 24hr* or 24 hour* or 24 hr* or continual* or continuous* or twenty four hour* or twenty four hr* or twentyfour hour* or twentyfour hr*).mp. (645505)  10 and/8-9 [Continuous & RRT] (21008)  11 CRRT*.tw,kf. (1381)  12 or/10-11 [CRRT concept] (21116)  13 Liver Failure/ (6831)  14 exp Liver Failure, Acute/ (5443)  15 exp Urea Cycle Disorders, Inborn/ (1681)  16 (fulmina* adj3 (hepat* or liver*)).tw,kf. (6809)  17 ((failure* or injur*) adj3 (hepat* or liver*)).tw,kf. (61849)  18 (hyper ammonaemia* or hyper ammonemia* or hyperammonaemia* or hyperammonemia*).tw,kf. (3503)  19 urea cycle disorder*.tw,kf. (681)  20 or/13-19 [Combined MeSH & text words for acute liver injury] (72569)  21 and/12,20 [Combined concepts for CRRT & liver injury] (489)  22 exp animals/ not humans/ (4524057)  23 (animal* or bovine* or calves or camel* or canine* or cat or cats or chimp* or dog or dogs or equine* or feline* or goat* or hamster* or horse* or llama* or mice* or monkey* or mouse* or pig or piglet* or pigs or porcine* or primate* or rabbit* or rat or rats or rodent* or sheep* or simian* or swine* or veterinar*).ti. (2206424)  24 21 not (22 or 23) [Exclude animal studies] (470)  25 limit 24 to yr="1990-Current" (456)  26 remove duplicates from 25 (456) |  |
| --- | --- |
